# PglB function and glycosylation efficiency is temperature dependent when the *pgl* locus is integrated in the *Escherichia coli* chromosome

**DOI:** 10.1186/s12934-021-01728-7

**Published:** 2022-01-05

**Authors:** Vanessa S. Terra, Marta Mauri, Thippeswamy H. Sannasiddappa, Alexander A. Smith, Mark P. Stevens, Andrew J. Grant, Brendan W. Wren, Jon Cuccui, the Glycoengineering of Veterinary Vaccines consortium (GoVV)

**Affiliations:** 1 London School of Hygiene and Tropical Medicine, Keppel Street, London, WC1E7HT UK; 2 Department of Veterinary Medicine, University of Cambridge, Madingley Road, Cambridge, CB3 0ES Cambridgeshire UK; 3 The Roslin Institute and Royal (Dick) School of Veterinary Studies, University of Edinburgh, Edinburgh, EH25 9RG UK

**Keywords:** Biological conjugation, PglB, Temperature, PGCT, One health, Poultry, Vaccine

## Abstract

**Background:**

*Campylobacter* is an animal and zoonotic pathogen of global importance, and a pressing need exists for effective vaccines, including those that make use of conserved polysaccharide antigens. To this end, we adapted Protein Glycan Coupling Technology (PGCT) to develop a versatile *Escherichia coli* strain capable of generating multiple glycoconjugate vaccine candidates against *Campylobacter jejuni*.

**Results:**

We generated a glycoengineering *E. coli* strain containing the conserved *C. jejuni* heptasaccharide coding region integrated in its chromosome as a model glycan. This methodology confers three advantages: (i) reduction of plasmids and antibiotic markers used for PGCT, (ii) swift generation of many glycan-protein combinations and consequent rapid identification of the most antigenic proteins or peptides, and (iii) increased genetic stability of the polysaccharide coding-region. In this study, by using the model glycan expressing strain, we were able to test proteins from *C. jejuni*, *Pseudomonas aeruginosa* (both Gram-negative), and *Clostridium perfringens* (Gram-positive) as acceptors. Using this *pgl* integrant *E. coli* strain, four glycoconjugates were readily generated. Two glycoconjugates, where both protein and glycan are from *C. jejuni* (double*-*hit vaccines)*,* and two glycoconjugates, where the glycan antigen is conjugated to a detoxified toxin from a different pathogen (single-hit vaccines). Because the downstream application of Live Attenuated Vaccine Strains (LAVS) against *C. jejuni* is to be used in poultry, which have a higher body temperature of 42 °C, we investigated the effect of temperature on protein expression and glycosylation in the *E. coli pgl* integrant strain.

**Conclusions:**

We determined that glycosylation is temperature dependent and that for the combination of heptasaccharide and carriers used in this study, the level of PglB available for glycosylation is a step limiting factor in the glycosylation reaction. We also demonstrated that temperature affects the ability of PglB to glycosylate its substrates in an in vitro glycosylation assay independent of its transcriptional level.

**Supplementary Information:**

The online version contains supplementary material available at 10.1186/s12934-021-01728-7.

## Background

Protein Glycan Coupling Technology (PGCT) exploits genetically modified bacteria to produce glycoconjugates, in essence transforming bacterial cells into vaccine production factories [1]. The technology obviates the need to handle pathogens and eliminates expensive and time-consuming purification steps as well as simplifying glycan-acceptor protein coupling [2]. Its conception stemmed from the discovery that protein glycosylation is present in all domains of life [3, 4] and is the most common post-translational modification of proteins. In the late 1990s an *N*-linked protein glycosylation locus (*pgl*) was identified in the bacterium *C. jejuni* [5]. This was followed by the functional transfer of the locus into a laboratory strain of *E. coli* with the first glycoprotein generated heterologously [6]. Subsequent transfer of the *pgl* locus demonstrated that in *E. coli*, the oligosaccharyltransferase (OST) PglB could transfer different polysaccharide structures to an acceptor protein [7]. Concomitantly to these findings, it was demonstrated that PglB preferred a consensus sequence (sequon) D/E-X_1_-N-X_2_-S/T, (where X_1_ and X_2_ cannot be a proline) [8]. The discovery of the consensus sequon combined with the relaxed specificity of PglB towards different polysaccharide regions has contributed to burgeoning advancements in the field of glycoengineering. In 2011 Fisher et al., demonstrated that the addition of a sequence consensus ‘glycotag’ at either the *N*-or *C*-termini of a protein was sufficient for glycosylation [9]. This discovery meant that double-hit approach vaccines (where the protein and glycan are from the same pathogen) are a possibility, as any protein can become an acceptor. Furthermore, it allows new glycoconjugate vaccines to overcome the use of the already overly utilised CRM197, tetanus toxoid (TT) and *Haemophilus influenzae* protein D [10] carrier proteins. The use of new carrier proteins contributes towards avoiding possible interference between different vaccines [11], carrier-induced immune suppression [12], as well as increasing vaccine specificity and coverage.

Despite recent technical developments, the need to improve PGCT as an alternative glycoconjugate vaccine production platform remains. In this work, we generated a versatile glycoengineering strain (*E. coli* SDB1*pgl*) for the rapid production of glycoconjugates to be used recombinantly as well as to investigate the usefulness of such strains as part of a live attenuated vaccine strategy. The *E. coli* SDB1*pgl* was used to investigate glycosylation in vivo in a bid to understand the lack of protection observed in our previous study where poultry was vaccinated using avian pathogenic *Escherichia coli* (APEC) strain Chi7122*pgl* [13]. By creating a strain that can chromosomally express the conserved *Campylobacter* heptasaccharide glycan we introduced greater flexibility to the PGCT system, enabling the rapid testing of preferred carrier proteins, obviating the need for antibiotics for plasmid maintenance, minimising the possible spread of plasmids and increasing stability of the polysaccharide coding region as well as contributing to greater efficiency by decreasing the metabolic burden to the *E. coli* host [14–17]. Here we show that with appropriate modifications, Gram-positive or Gram-negative bacterial proteins could be modified with *C. jejuni* glycan in *E. coli* SDB1 cells [18]. The *pgl* locus is an optimal starting point as it contains both the necessary genes for the assembly of the *C. jejuni* heptasaccharide as well as the PglB OST. We demonstrated that by using an *E. coli pgl* integrant strain, we can rapidly generate different recombinant glycoconjugates as well as potentially produce glycoconjugates within the bacteria that, after the introduction of mutations to reduce toxicity, could be used as a delivery method for vaccines. Due to results we have observed previously, where no protection was seen against *C. jejuni* when using Chi7122*pgl,* we set out to determine factors that affect glycosylation when the *pgl* locus is inserted in the chromosome. As the live attenuated vaccines (LAVs) created in our previous study [13] were intended to be used in poultry which have a higher body temperature than humans or other farm animals [19], we investigated the effect of temperature on protein expression and glycosylation. An unequivocal relationship between temperature and glycosylation efficiency was demonstrated within bacterial cells as well as when carrying out the reaction using an in vitro glycosylation assay. This alteration in glycoconjugate production was shown to be independent of gene expression at the mRNA level. Finally, we determined that the addition of extra copies of *pglB* per cell improves glycosylation efficiency under the conditions tested.

## Results

### Generation of the SDB1*pgl* integrant

To establish a flexible system for the rapid generation of glycoconjugate vaccines against *Campylobacter*, we decided to insert the *C. jejuni pgl* locus into the chromosome of the *E. coli* SDB1 strain via allelic exchange. *E. coli* SDB1 [18] is a W3110-derivative strain lacking the O-antigen with the additional deletion of *waaL* and *wecA* genes encoding the O-antigen ligase and the initiating glycosyltransferase involved in O-antigen and enterobacterial common antigen (ECA) biosynthesis, respectively. This glycoengineering strain was chosen to maximise the amount of glycan substrate available for PglB (owing to the absence of competition with the WaaL ligase) and to ensure the faithful synthesis of *C. jejuni* heptasaccharide starting with the correct reducing end glycan, bacillosamine, transferred by the PglC initiating transferase instead of a mixture of GlcNAc-starting glycans initiated by the *E. coli* endogenous initiating transferase WecA.

**Fig. 1:**
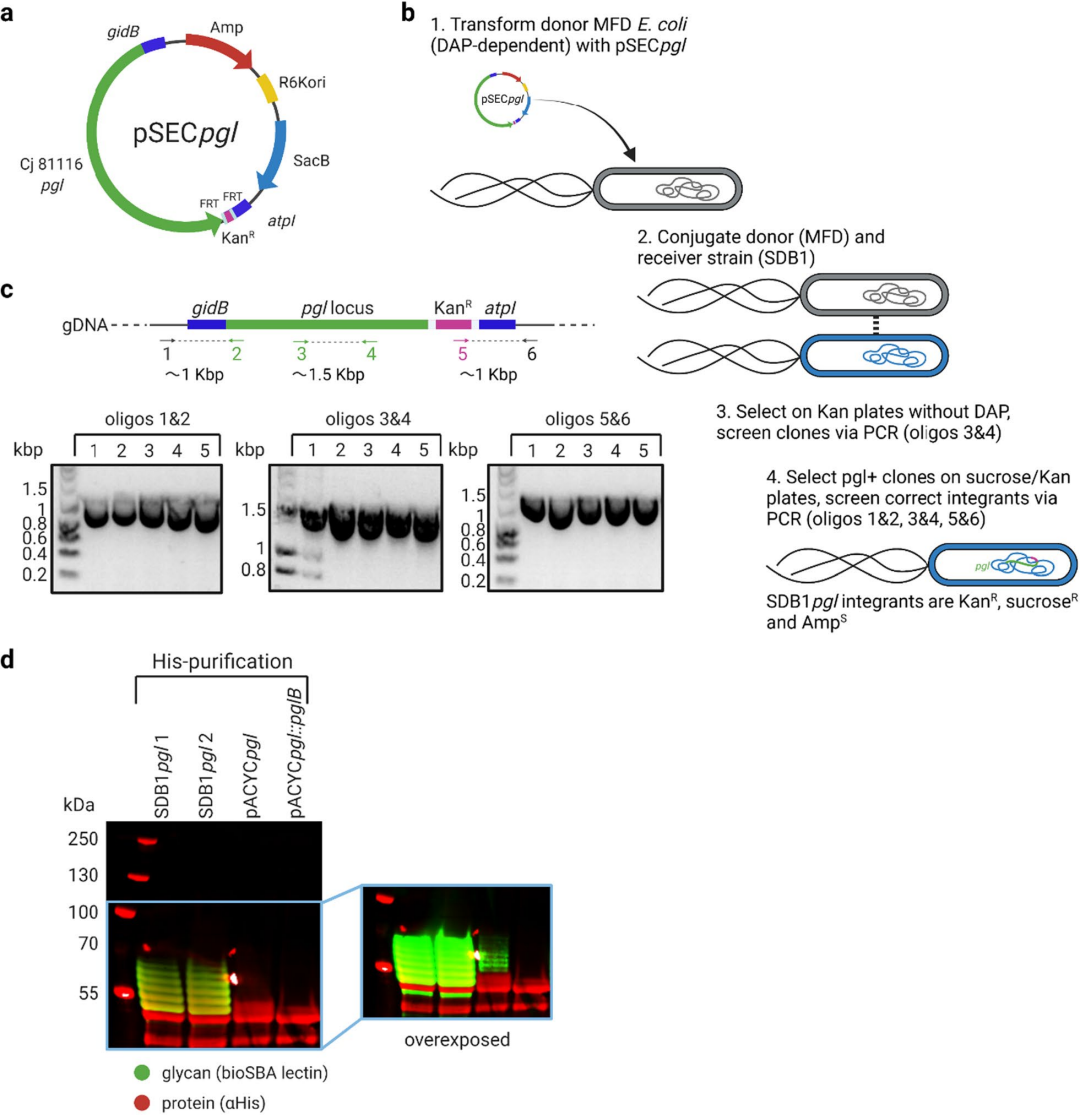
Generation of functional SDB1pgl integrants. **A** Schematic diagram depicting the pSEC*pgl* suicide plasmid. **B** Strategy for integration of the *C. jejuni pgl* locus in the SDB1 genome via allelic exchange. **C** Confirmation of correct integration of the *pgl* locus via PCR in five SDB1*pgl* clones. Oligos 1 and 6 align outside of the chosen homology arms, while oligos 2 and 5 align to the *pgl* locus and the KanR tag, respectively. Formation of amplicons 1&2 and 5&6 is possible only in case of successful integration in the correct location. **D** SDB1*pgl* integrants can glycosylate a model carrier protein (G-NetB). SDB1 transformed with a plasmid encoding *pgl* locus (pACYC*pgl*) as well as a plasmid encoding *pgl* locus with a non-functional PglB (pACYC*pgl*::*pglB*) were used as positive and negative controls, respectively. His-pulldown followed by western blotting confirms glycoprotein production

To achieve chromosomal integration of the *pgl* locus, we used the suicide vector, pSEC*pgl* [13] based on the pCVD442 plasmid [20] containing a *pir*-dependent R6K replicon and the *sacB* gene of *Bacillus subtilis* conferring sucrose sensitivity, which enables positive selection of integrants for the loss of backbone sequence. pSEC*pgl* contains the *pgl* locus from *C. jejuni* 81116 C-terminally tagged with a kanamycin resistance (KanR) cassette and surrounded by homology arms for insertion between *E. coli gidB* and *atpI* genes (Fig. 1A).

pSEC*pgl* was delivered to recipient SDB1 cells via overnight conjugation with DAP-dependent MFD donor cells (Fig. 1B). Integrants were identified by growth on selective agar and confirmed by PCR. A successful first recombination event was identified by kanamycin-resistant and DAP-sensitive conjugants that were positive for a PCR amplifying the middle of the *pgl* locus with oligonucleotides 3&4 resulting in a ∼1.5 kb amplicon (step 3 in Fig. 1B).

To test whether the SDB1*pgl* integrants possessed a functional *pgl* locus, two clones were transformed with an IPTG-inducible plasmid encoding a hyperglycosylatable version of *C. perfringens* necrotic enteritis toxin (G-NetB). Both clones of SDB1*pgl* integrants tested were functional as they clearly glycosylated G-NetB (Fig. 1D). Six bands (green) corresponding to G-NetB glycosylated at one to six sites are visible by fluorescent western blotting when stained with SBA lectin, which recognises the terminal GalNAc residue of the *C. jejuni* heptasaccharide. In this specific example, the integrants outperformed the very same strain transformed with a plasmid encoding the *pgl* locus (pACYC*pgl*, ∼10–12 copies per cell), suggesting that, at least in this case, a single chromosomal copy of *pgl* in concert with the expression of G-NetB from a pEXT20 backbone provides more efficient glycoconjugate production. No glycosylation could be observed when SDB1 was transformed with a plasmid encoding a version of the *pgl* with a mutation in *pglB* (pACYC*pgl*::*pglB*), which served as a negative control.

### Generation of vaccine candidates using SDB1*pgl* and SDB1pACYC*pgl* host strains

**Fig. 2:**
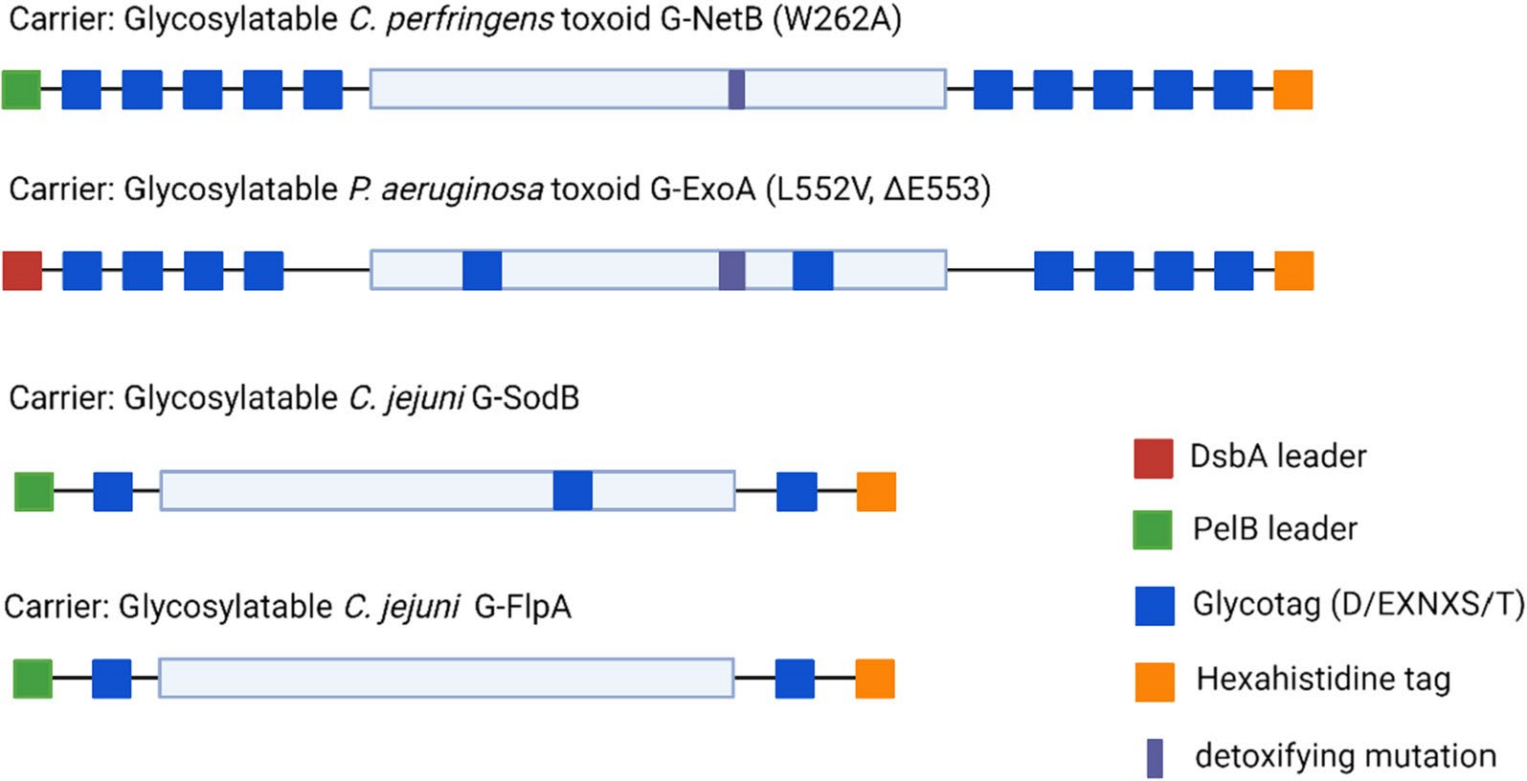
Depiction of the modifications performed in the carrier proteins to become suitable PglB substrates (addition of different numbers of non-natural glycotags (2, 8 and 10), PelB or DsbA leaders for co-localisation in the periplasm, and poly-histidine tag for purification as well as detoxifying mutations on both G-ExoA and G-NetB)

Most proteins are not natural substrates for the OST PglB, however with modifications a protein can be decorated with a PglB-compatible polysaccharide (Fig. 2). For this purpose, we modified G-NetB, G-ExoA, G-SodB and G-FlpA by adding glycotags, a signal peptide, either PelB or DsbA, for localisation to the periplasmic compartment and a polyhistidine tag for affinity purification of the glycoconjugates. All protein modifications are described in “Materials and methods” and are summarised in Fig. 2.

### Comparison of glycosylation of carrier proteins between SDB1*pgl *and SDB1 pACYC*pgl* at 30 °C and 37 °C

**Fig. 3:**
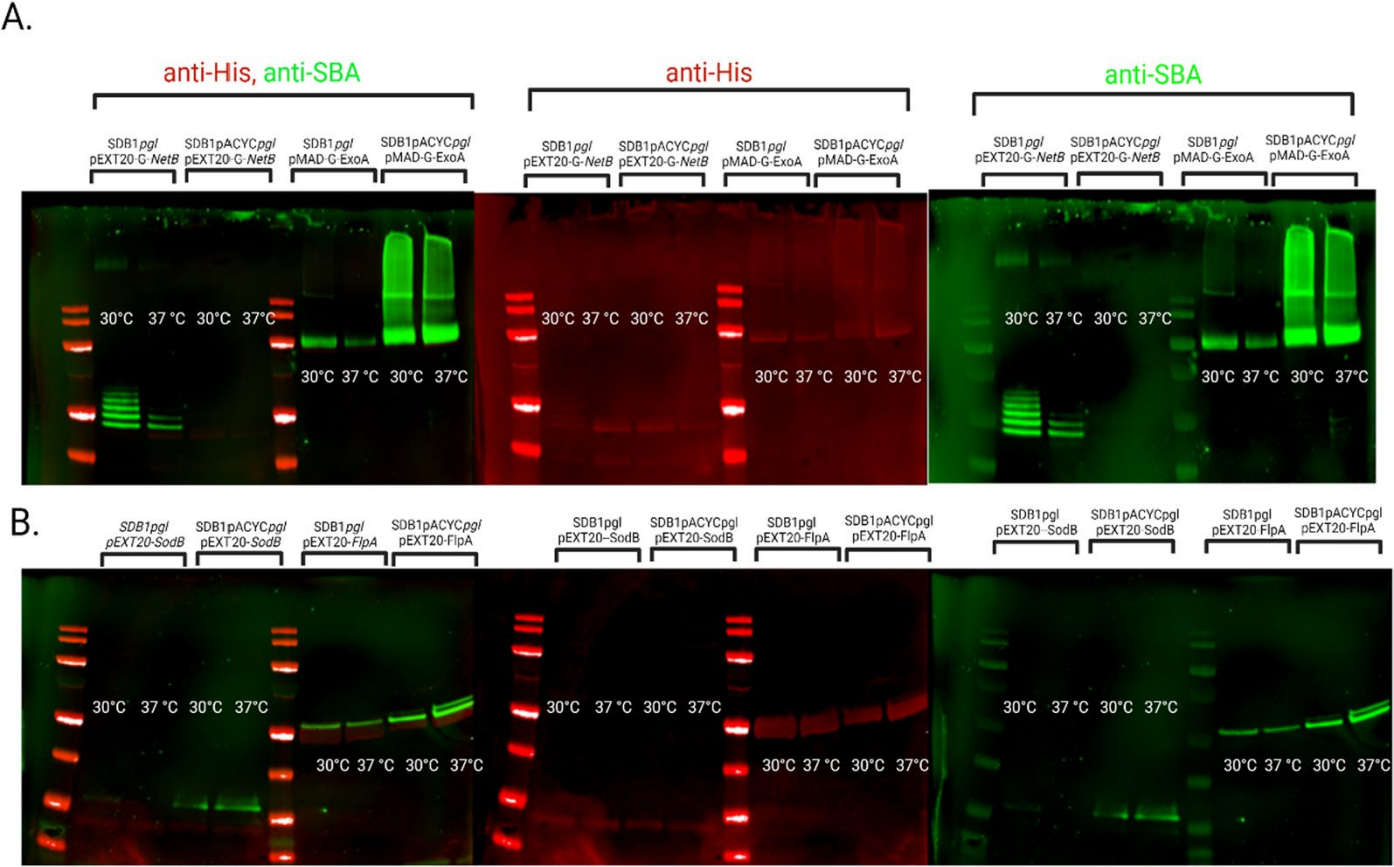
Comparison of glycosylation of four different carriers NetB and G-ExoA (**A**) and G-SodB, G-FlpA (**B**), by SDB1pgl, which expresses the *C. jejuni* heptasaccharide from its chromosome and SDB1 pACYCpgl, which expresses the *C. jejuni* heptasaccharide from a pACYC184-based plasmid, at two different temperatures (30 °C and 37 °C). In red is the anti-His signal and in green the anti-glycan signal

The need to understand how different factors such as temperature, media and co-factors affect glycosylation and to determine optimal conditions to standardise PGCT has been an imperative goal. However, glycosylation variability has been observed and reported, depending on the glycan or the carrier protein used. To determine the effects that different copy numbers of the *pgl* glycosylation locus and different temperatures have on glycoconjugate formation, we compared the newly generated *E. coli* SDB1*pgl* strain with a single chromosomal copy of the *pgl* locus, to an *E. coli* SDB1 expressing the *pgl* locus from plasmid pACYC*pgl* (SDB1 pACYC*pgl*∼10–12 copies per cell) and assessed qualitatively the variability of glycosylation of carriers G-SodB, G-FlpA, G-NetB and G-ExoA at 30 °C and 37 °C (Fig. 3).

We observed that for three of the four proteins tested (G-FlpA, G-ExoA and G-SodB) glycosylation was evidently higher with the *pgl* locus residing on a plasmid (Fig. 3). Protein production for G-FlpA was similar for SDB1*pgl* and for SDB1 pACYC*pgl* at 30 °C (Table 1) however, its expression was markedly different at 37 °C (SDB1*pgl*_pEXT20-FlpA 37 °C—1.09 mg/ml and SDB1 pACYC*pgl*_pEXT20-FlpA 0.41 mg/ml). Even though, protein quantity produced in the *E. coli* cells containing the *pgl* locus in the chromosome (SDB1*pgl*) was 2.65 times higher than the protein produced in the *E. coli* cells expressing the *pgl* locus from the plasmid, the glycosylation level was markedly different, with only one sequon occupied in SDB1*pgl*_pEXT20-F*lpA* at 37 °C as opposed to two in SDB1pACYC*pgl_*pEXT20-FlpA at the same temperature.

**Table 1:** Acceptor proteins quantities produced in the same *E. coli* SDB1 background but with the *pgl* locus in plasmid (SDB1 pACYC*pgl*) or inserted in the chromosome of SDB1 (SDB1*pgl*) at 30 °C and 37 °C (X indicates ORF inserted coding for G-SodB/G-FlpA/G-NetB/G-ExoA)

Temperature (°C)	*E. coli* background	Acceptor proteins			
		G-SodB	G-FlpA	G-NetB	G-ExoA
30	SDB1 pACYC*pgl* pEXT20_X	0.4	0.92	0.40	1.79
	SDB1*pgl* pEXT20_X	0.24	0.90	0.28	0.83
37	SDB1 pACYC*pgl* pEXT20_X	0.54	0.41	0.28	0.71
	SDB1*pgl* pEXT20_X	0.45	1.09	0.28	0.55

G-SodB was expressed similarly at both temperatures and by both bacterial hosts (Table 1). However, glycosylation was visibly better for the plasmid-based system suggesting that protein quantity is not directly related to glycosylation efficiency (Fig. 3). There was greater protein production for SDB1pACYC*pgl*_pMAD-G-ExoA than SDB1*pgl_*pMAD-G-ExoA at both temperatures (Table 1). There was a clear difference on the glycosylation pattern observed for SDB1pACYC*pgl*_pMAD-G-ExoA when compared with SDB1*pgl_*pMAD-G-ExoA. Glycosylation was clearly more efficient when the *pgl* locus was in a plasmid (SDB1pACYC*pgl*_pMAD-G-ExoA) as opposed to inserted in the chromosome (SDB1*pgl_*pMAD-G-ExoA).

The fourth protein tested G-NetB was unique in its phenotype since it was the only one modified more efficiently when the *pgl* locus was inserted in the chromosome. At 30 °C, six out of its ten sequons were glycosylated as opposed to two at 37 °C. Interestingly there were small differences in protein quantities produced using the different hosts, SDB1pACYC*pgl*_pEXT20-G-NetB (0.40 mg/ml) and SDB1*pgl*_pEXT20-G-NetB (0.28 mg/ml) at 30 °C (Table 1). SDB1pACYC*pgl*_pEXT20-G-NetB and SDB1*pgl*_pEXT20-G-NetB both produced same quantity of protein at 37 °C (0.28 mg/ml) (Table 1). In this instance there were fine differences between protein quantities, however SDB1pACYC*pgl* pEXT20-G-NetB produced a higher amount of protein at 30 °C and this did not correlate positively with more efficient glycosylation (Fig. 3, panel A). The glycosylation deficiency observed for three out of the four proteins tested in the SDB1*pgl* strains led to the hypothesis that PglB might be limiting the glycosylation reaction since *pglB* was being expressed constitutively from a single genomic copy as opposed to the expected 10–12 copies per bacterial cell from the pACYC*pgl* plasmid.

### Generation of glycoconjugates using SDB1*pgl* with additional inducible PglB

**Fig. 4:**
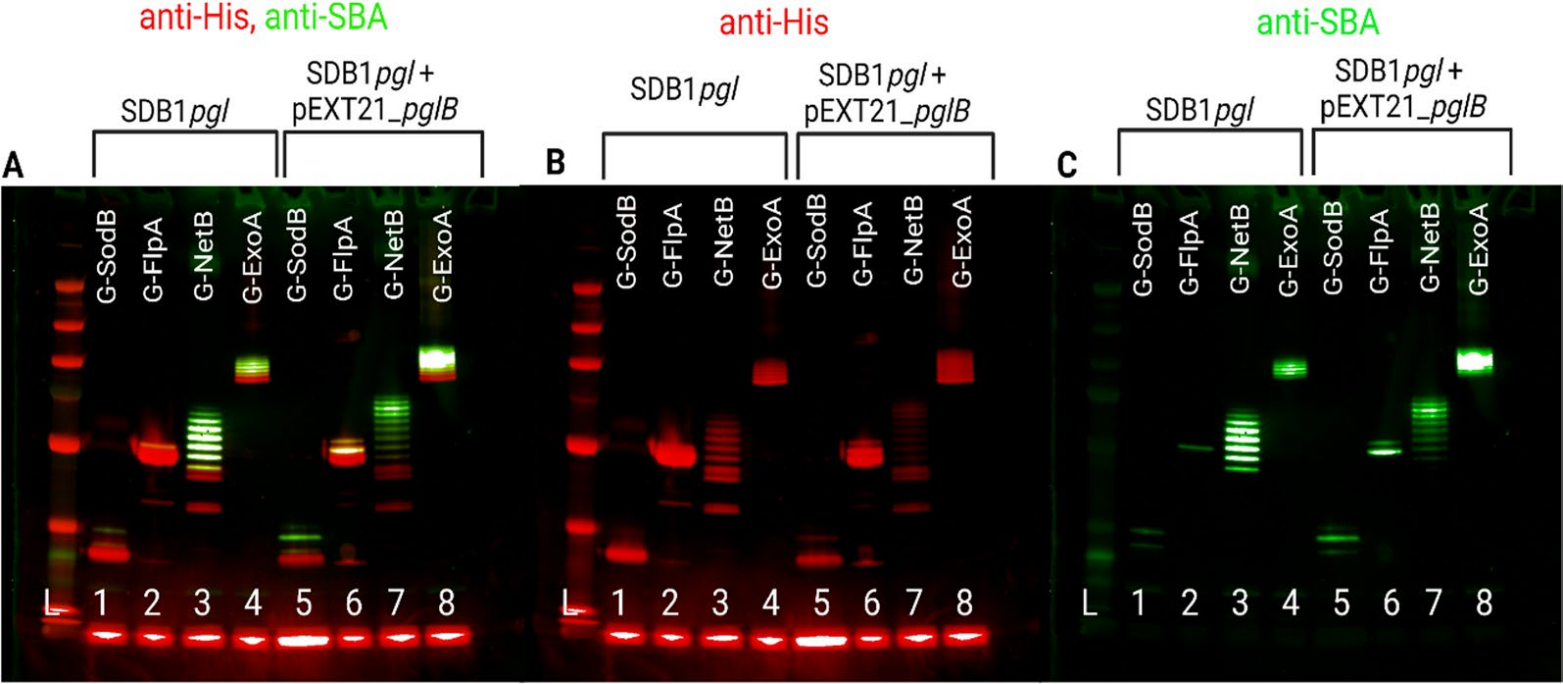
Comparison between glycosylation of carrier proteins at 30 °C in the presence and absence of additional inducible PglB. Glycosylation without additional inducible PglB of carriers G-SodB (lane 1), G-FlpA (lane2), G-NetB (lane 3) and G-ExoA (lane 4), followed by the effect of addition of inducible PglB in glycosylation of the same carriers at 30 °C, G-SodB (lane 5), G-FlpA (lane 6), G-NetB (lane 7) and G-ExoA (lane 8). 2 µg of each protein was loaded per well. In red is the anti-His and in green the anti-SBA signals

To test if PglB and not the available heptasaccharide was the limiting factor on three out of four of the glycosylation reactions using the chromosomally inserted *pgl* locus, an additional IPTG inducible plasmid-borne *pglB* was electroporated into strains SDB1*pgl* pEXT20-SodB, SDB1*pgl* pEXT20-FlpA, SDB1*pgl_*pEXT20-G-NetB and SDB1*pgl_*pG-ExoA generating strains SDB1*pgl*_pEXT21_*pglB*_pEXT20-SodB, SDB1*pgl*_pEXT21*pglB*_pEXT20-FlpA, SDB1*pgl*_pEXT21_*pglB*_pEXT20-G-NetB and SDB1*pgl*_pEXT21*pglB*_*p_*G-ExoA. The plasmid backbone encoding PglB is pEXT21, which has an estimated copy number of ∼3 per cell [23], thereby increasing the total *pglB* copy number to 4 (one constitutively expressed from the endogenous promoter of the *pgl* locus and three from a P_*tac*_ IPTG-inducible promoter). Glycosylation efficiency was tested in these strains at 30 °C, the temperature previously identified as most favourable for glycosylation. The glycosylation efficiency improved considerably for carriers G-SodB (lane 1 and lane 5), where it is clearly visible that both sequons are occupied, as well as for G-NetB where all 10 sequons are occupied (lane 7), i.e., 3 more than without the extra inducible PglB (Fig. 4, lane 3). A ladder pattern as well as more intense bands could be observed for G-ExoA when we probed with SBA lectin (Fig. 4, lane 8), which is an improvement from the result obtained without the addition of the extra inducible PglB, lane 4). Furthermore, with the addition of inducible PglB we could observe an increase in glycosylation of FlpA with both sequons occupied (lane 5) as opposed to just one (lane 2). These observations led to the conclusion that efficient glycosylation of the protein carriers tested need more than one functional copy of *pglB* per cell, suggesting that once the polysaccharide coding region is integrated in the chromosome, *pglB* can be a limiting factor for the glycosylation reaction for these acceptors and not glycan production.

### Glycosylation of all four carriers at 42 °C using SDB1*pgl* and SDB1*pgl_*pEXT21*pglB*

The idea of using bacterial cells as a vector for glycoconjugate delivery is a concept that is gaining traction in the veterinary field, particularly if the bacterial cells producing the vaccine can be added to the water or the feed of the target animal for ease of administration at scale.

**Fig. 5:**
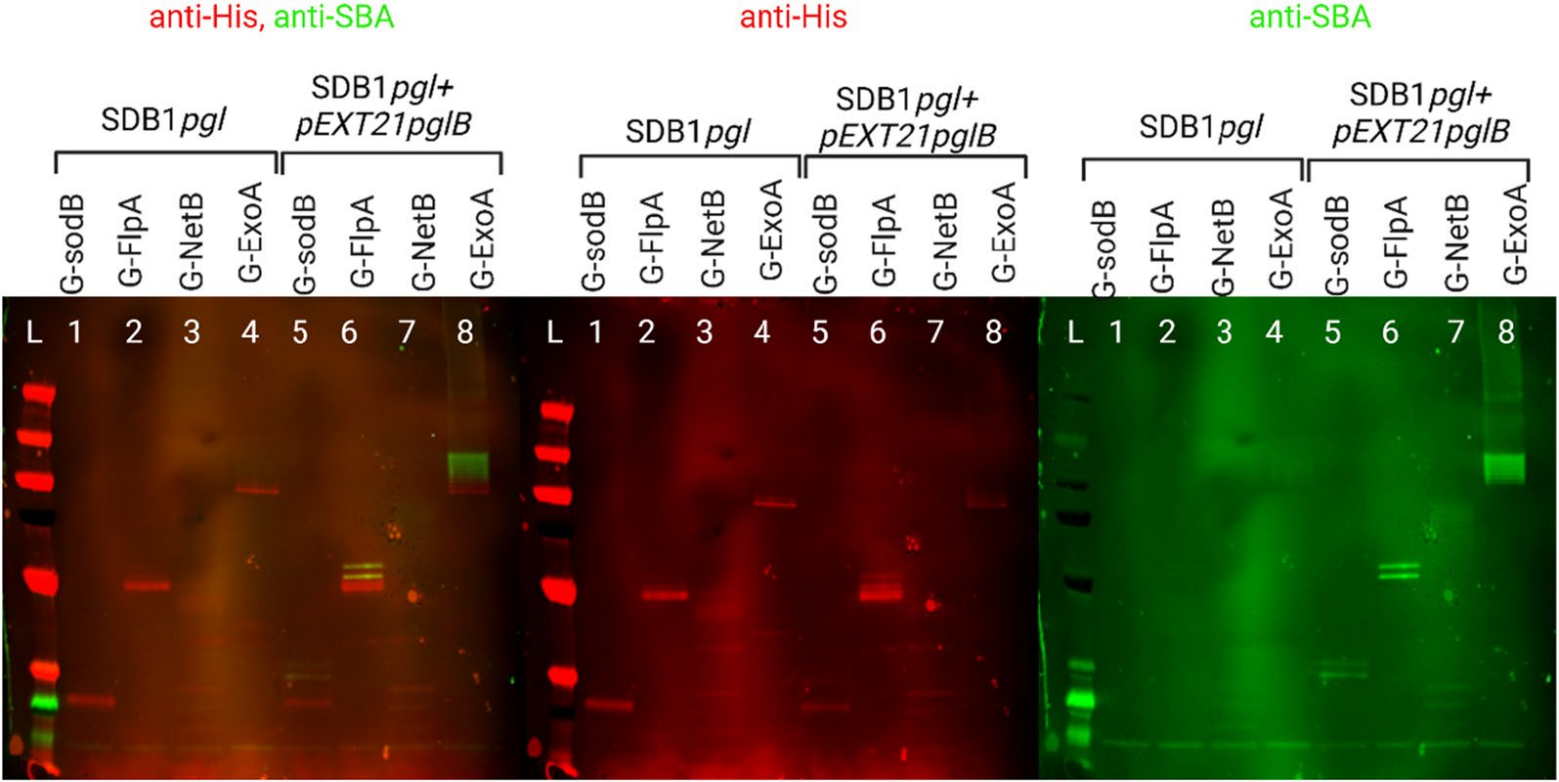
Glycosylation of carrier proteins at 42 °C in SDB1*pgl* and SDB1*pgl* _pEXT21*pglB* backgrounds. Lanes, 5, 6, 7 and 8 show the increase in glycosylation potentiated by the addition of extra inducible PglB. Lanes 1, 2, 3 and 4 show lack of detectable glycosylation at 42 °C in SDB1*pgl* background. In red is the anti-His signal and in green the anti-glycan signal

Due to the glycosylation temperature dependency that we report in this manuscript, and the fact that we have been working towards a *C. jejuni* vaccine for chickens to reduce downstream campylobacteriosis in humans, we decided to investigate glycosylation efficiency at 42 °C, the approximate body temperature of chickens (Fig. 5). We observed a substantial decrease in glycosylation at 42 °C even when plasmid-borne inducible *pglB* was added when compared to glycosylation at 30 °C (Fig. 4). However, the effect of adding inducible PglB was still visible (Fig. 5, lanes 5, 6, 7 and 8) when compared to the chromosomally integrated *pgl* locus (SDB1*pgl*) only (Fig. 5, lanes 1 to 4). Interestingly, G-FlpA was the only protein to register an improvement in its glycosylation pattern. At this temperature and with the extra inducible PglB, both sequons were occupied (Fig. 5, lane 6).

**Fig. 6:**
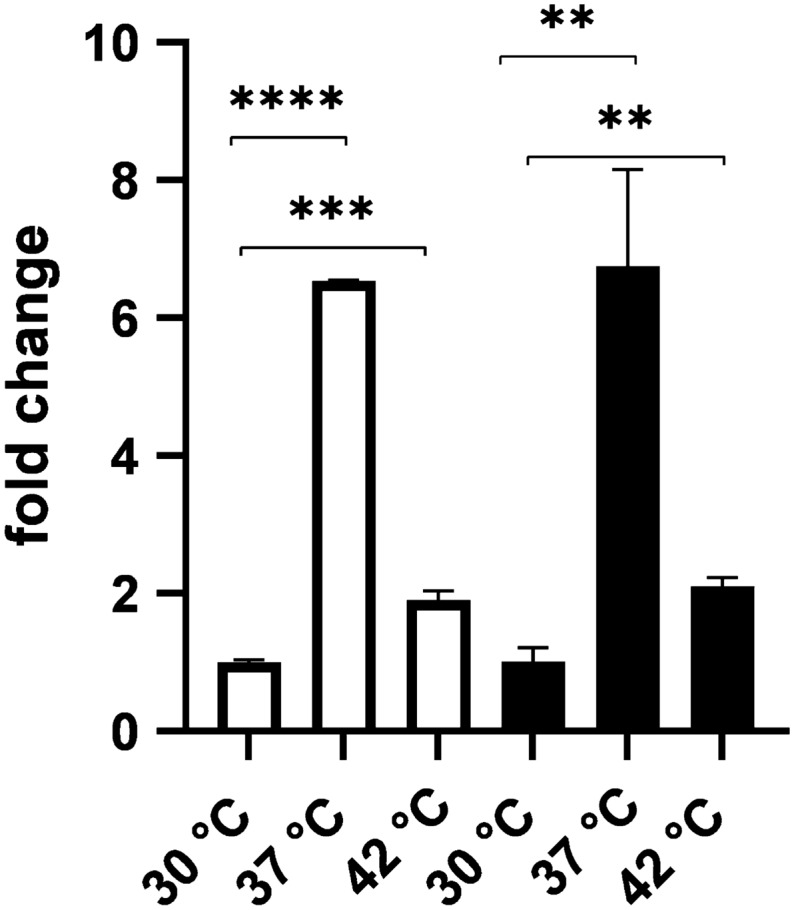
Transcription of *pglB* in SDB1*pgl* grown at 30 °C, 37 °C and 42 °C. In white *pglB* expression in SDB1*pgl* when compared to housekeeping gene *gapA*. In black *pglB* expression in SDB1*pgl* when compared to the housekeeping gene *rpoS*. Data was obtained using 3 biological replicates each with 3 technical replicates for each temperature (n = 9), p < 0.001

### *pglB* expression at 30 °C, 37 °C and 42 °C

To understand if the decrease in glycosylation observed at higher temperatures was a consequence of altered gene expression, transcription of *pglB* was analysed. Using quantitative RT-PCR and the Δ∆Ct [22] method we assessed the transcription of *pglB* compared to the housekeeping genes *gapA and rpoS* at the same three temperatures. Transcription of *pglB* at 30 °C was determined as the control condition since this is the temperature where the highest glycosylation of the carriers is observed. We observed an increase in the transcription of *pglB* at both 37 °C and 42 °C, of approximately sixfold and twofold (n = 9) respectively. However, this apparent increase in transcription of *pglB* does not appear to correlate with higher glycosylation of the carrier proteins (Fig. 6).

### In vitro glycosylation at 30 °C, 37 °C and 42 °C

**Fig. 7:**
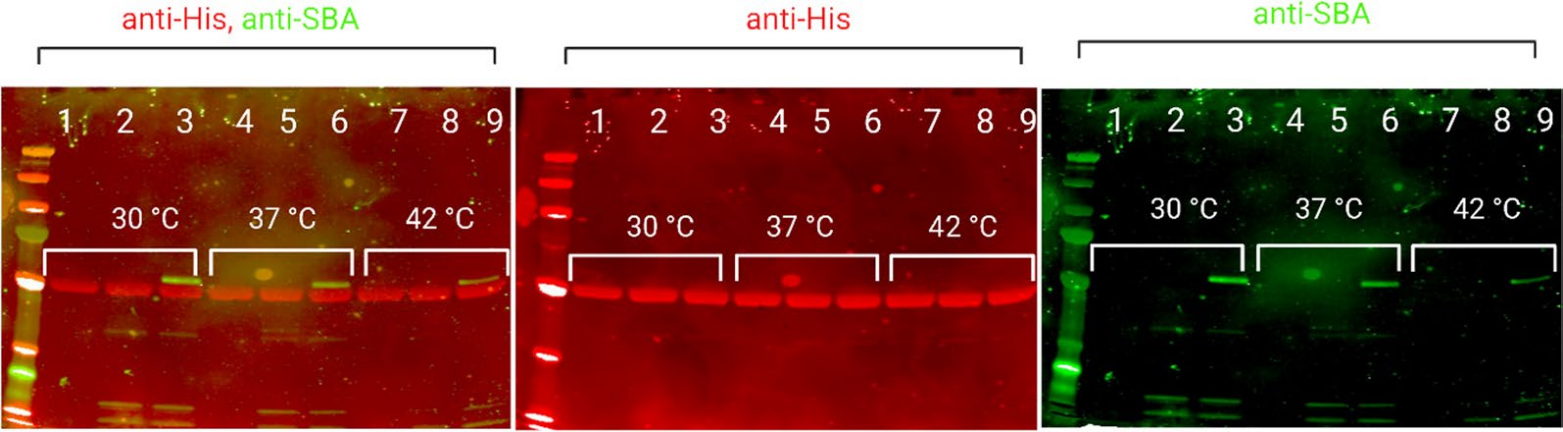
In vitro glycosylation reaction using carrier protein G-FlpA. L-Protein ladder PageRuler plus (Bioline, UK) Lane 1, 4 and 7—negative control, G-FlpA, no glycan donor incubated at 30 °C, 37 °C and 42 °C respectively; lane 2,5 and 8—G-FlpA combined with glycan donor from SDB1pACYCpgl and SDB1pEXT21pglB, incubated at 30 °C, 37 °C and 42 °C respectively; lane 3, 6 and 9—G-FlpA combined with glycan donor from SDB1pgl and SDB1pEXT21pglB incubated at 30 °C, 37 °C and 42 °C respectively

To try and understand the decrease in glycosylation at higher temperatures despite the increase in *pglB* expression, an in vitro glycosylation reaction was performed. For this assay all bacterial cell extracts were prepared at 30 °C, all the SDB1pEXT21*pglB* expressing *E. coli* was prepared in a single batch culture, ensuring the same level of PglB per reaction. The incubation of the reaction was then performed at 30 °C, 37 °C and 42 °C. In vitro glycosylation reactions had varied degrees of success depending on the carrier protein used. Using carrier protein FlpA we were able to determine a clear effect of temperature on the function of PglB. It was clear that the reactions at the higher temperatures of 37 °C and 42 °C had significantly less glycan being detected by SBA lectin (Fig. 7, lanes 6 and 9). G-NetB degraded when incubated in the in vitro reaction conditions while G-SodB presented a similar phenotype to FlpA, with glycosylation decreasing as the reaction temperature increases (Additional file 1: Fig. S1). G-ExoA was modified with the heptasaccharide to a very low level at 30 °C and below the detection level at the higher temperatures (Additional file 1: Fig. S2).

## Discussion

Understanding glycosylation variability is central for increasing the applicability of PGCT for vaccine development. Currently, most glycoconjugates generated using PGCT, also referred to as biological conjugation, use a plasmid-based system [23–31]. Despite it being successful when the goal is to generate recombinant glycoconjugates, reducing the number of plasmids in the system and increasing genetic stability of PGCT elements is desirable for vaccine process development. There are clear advantages to have the polysaccharide coding region integrated in the bacterial chromosome. Firstly, issues regarding plasmid incompatibilities and plasmid stability are avoided. Secondly, chromosomal integration of the glycan-encoding locus reduces the need for antibiotic use in the cultures to maintain extrachromosomal copies of plasmids. Importantly, flexibility is introduced in the system. By keeping the polysaccharide region constant one can readily assess multiple carrier proteins in terms of glycosylation efficiency and yield, overall enabling the more rapid production and testing of candidate vaccines in either a recombinant form or to be used as a future LAV. Introduction of large polysaccharide coding regions in the chromosome of bacteria has been shown as enabling stable and metabolically efficient glycan production [14, 15, 32]. Furthermore, having multiple copies of two or three plasmids performing complex metabolic functions can elevate the metabolic burden arresting cell growth [13, 33], having a deleterious effect on glycoconjugate production.

In this study we aimed to compare the expression and glycosylation of G-SodB, G-FlpA, G-NetB and G-ExoA when the polysaccharide is expressed from the chromosome versus expression of the same polysaccharide from plasmid pACYC*pgl* at different temperatures. Despite the metabolic burden resulting from expressing the entire *pgl* locus in the medium copy plasmid pACYC*pgl* (10–12 copies), three out of four of the protein carriers tested exhibit better glycosylation in this combination (G-SodB, G-FlpA, G-ExoA). We did observe, however, that for both methods a lower culture temperature (30 °C) promoted more efficient glycosylation for three out of the four acceptors tested (G-SodB, G-NetB and G-ExoA). Interestingly, it was only G-FlpA, a *C. jejuni* native protein, that showed better glycosylation at 37 °C with the plasmid system, even if the protein level produced was approximately 3 times inferior to the protein produced by SDB1*pgl.* In this case a higher protein yield did not correlate with higher glycosylation, which could even indicate a potential feedback inhibitory effect. There was also variability of sequon occupation. It was noted that both sequons added to G-FlpA were occupied when the protein was glycosylated in the presence of more than one copy of *pglB* at 30 °C, 37 °C and 42 °C. On the other hand, for protein G-NetB the opposite was true, higher temperature led to lower sequon occupancy in SDB1*pgl*. No noticeable glycosylation was observed when G-NetB was in the presence of multiple copies of the *C. jejuni* heptasaccharide. Therefore, for this carrier protein, extra availability of donor glycan and OST did not positively correlate with higher yield of glycosylated protein. Furthermore, we concluded based on the G-NetB result that using the plasmid system for screening for glycosylated proteins may not always be suitable for highly and efficiently glycosylated proteins. Why such pronounced effects were observed for G-FlpA and G-NetB needs further investigation. However, one can speculate that it could be related to the coupling rate of PglB/ carrier/ glycan and the availability of substrates at a given time. Low glycosylation efficiency in SDB1*pgl* was intriguing and led us to hypothesise that one copy of PglB per cell may not be sufficient for efficient glycosylation. We tested our hypothesis by adding an inducible low copy plasmid (3 copies per cell ± 0.3) [23] expressing PglB to SDB1*pgl*. We observed a substantial increase on occupation of sequons and therefore glycosylation, on all carrier proteins tested at 30 °C. The addition of only extra *pglB* copy but not all the other necessary components for the expression of the *C. jejuni* heptasaccharide, indicated that enough *C. jejuni* heptasaccharide was being produced in SDB1*pgl*, but that one *pglB* copy per cell was insufficient to efficiently transfer it across to the different acceptor proteins.

Because of our interest in applying PGCT to LAVS and of the temperature dependence of glycosylation reported in this manuscript, we have also assessed glycosylation of all four carriers at 42 °C, as it is the reported avian body temperature [19]. We have observed that glycosylation still happens at 42 °C but it is not efficient (Fig. 5). To ensure that the decrease seen in glycosylation was not due to a decrease in *pglB* expression we investigated the fold-change of *pglB* expression at 30 °C, 37 °C and 42 °C and observed a fold-increase of approximately 6 and 2 respectively. However, an increase in the expression of *pglB* did not result in higher glycosylation of the carrier proteins. These results were followed by the investigation of the effect of the temperature in the function of PglB by using a modified version of the in vitro glycosylation reaction described by Jaroentmeechai et al., [21]. In this instance, and due to the reaction conditions, different levels of glycosylation success were observed for the protein carriers studied. It is important to understand that this assay is performed using lysed bacterial cell cultures with no addition of protease inhibitors and at temperatures that potentiate protein degradation. For example, G-NetB was unable to withstand these conditions and degraded in all three temperatures tested.

The different glycosylation efficiencies observed in the in vitro glycosylation reaction for G-SodB, G-FlpA G-NetB and G-ExoA were not surprising as even in vivo glycosylation of these four proteins shows variation. Successful G-FlpA glycosylation using the in vitro glycosylation reaction best illustrates the effect of temperature on PglB function (Fig. 7). G-FlpA was modified at all three temperatures, however the fluorescence detected by the LICOR imaging system clearly shows a decrease as the reactions were incubated at higher temperatures.

In this study we have established a correlation between temperature and glycosylation yield for 3 out of the 4 proteins tested using the plasmid system, whereby glycosylation is more efficient at 30 °C than at 37 °C even when the protein level produced is similar. We also determined that glycosylation decreases for all proteins tested at 42 °C, and that this is independent of *pglB* expression. Thus, indicating an effect of temperature on PglB function. The observed temperature dependence of glycosylation could have more significant implications on the development of LAVS expressing glycoconjugates. It would mean the determination of a minimum amount of glycoconjugate that confers protection and ensuring this could be achieved per bacterial cell. Indeed, it may explain the lack of protection observed with an APEC *pgl* integrant expressing G-NetB in our recent work [13]. Alternatively, LAVS could be produced at 30 °C, and repeatedly introduced to chickens over time. Because the temperature effect seems to be on PglB function and independent of impacts on *pglB* transcription, adding a *pglB* chromosomal copy, possibly under the control of a constitutive promoter, would not be expected to increase glycosylation to the level necessary per bacterial cell.

It was surprising to observe such direct correlation between temperature and PglB function, as this protein is native to *C. jejuni*—a bacterium well adapted to the chicken’s higher body temperature. This temperature effect was observed both in vitro and in vivo in *E. coli* suggesting that perhaps there are other unknown factors that help stabilise PglB function in *C. jejuni*. Questions that remain unanswered and are under investigation include whether PglB requires additional help from a chaperone, for instance, to perform its function; and whether protein folding and sequon accessibility are affected by the temperatures at which cultures are grown.

Overall, it was observed that glycosylation is more efficient when there are more copies of *pglB* available and at lower temperatures. However, *pglB* does not have to be expressed from a high-copy number plasmid, instead the addition of an inducible *pglB* on a low copy number plasmid was sufficient. Furthermore, there was no positive correlation between the amount of protein produced by the bacterial cell and its glycosylation level, in fact the opposite was consistently observed for G-FlpA. A fundamental understanding of PglB when expressed in *E. coli* in the context of biological conjugation is central to the vaccine manufacturing process and its increased applicability.

## Conclusions

In this study we have established a correlation between temperature and glycosylation yield when we used the plasmid system, whereby glycosylation is more efficient at 30 °C than at 37 °C even when the protein level produced is similar. Furthermore, we have determined that for more efficient glycosylation when the *pgl* locus is integrated in the chromosome of SDB1 and at 30 °C, there is a need to have more than one copy of PglB available. This was true for all carrier proteins tested. Additionally, we determined that glycosylation decreases for all proteins tested at 42 °C, and that this is independent of *pglB* expression. Thus, indicating an effect of temperature on PglB function.

## Materials and methods

### Bacterial strains and growth conditions

*Escherichia coli* SDB1 (W3110, Δ*waaL* ligase, Δ*wecA* GlcNAc transferase), acquired from the Feldman laboratory [18] was used in this study. Consistently, *E. coli* was grown in Luria Bertani Lennox (LB) broth or LB agar plates at 30 °C, 37 °C and 42 °C. Where appropriate, kanamycin (50 μg/ml), ampicillin (100 μg/ml), chloramphenicol (30 μg/ml) and spectinomycin (100 μg/ml) were added to the culture medium.

### Generation of SDB1*pgl* integrants

SDB1 *pgl* integrants were generated via allelic exchange mediated by pSEC*pgl* suicide plasmid (Table 2) [13]. The pSEC*pgl* suicide vector (Amp^R^) was first transformed into electrocompetent diaminopimelic acid (DAP)-dependent *E. coli pir* + MFD donor cells [34] suitable for conjugation. Successful transformants were selected on LB agar plates supplemented with 0.3 mM DAP and 100 µg/ml Amp. 5 ml cultures of donor (MFD) and recipient (SDB1) strains were grown overnight at 37 °C and 180 rpm. The donor strain was grown in the presence of DAP (0.3 mM) and ampicillin (100 μg/ml) to maintain the suicide plasmid. Overnight cultures were diluted 1:100 in fresh LB medium with appropriate antibiotics. When cultures reached OD_600nm_ of 0.6–0.8 they were centrifuged for 2 min at 10,000×*g*. The bacterial pellets were then washed in PBS three times followed by resuspension in 100 ml fresh LB medium without antibiotics. The bacterial cells were then combined in a 1:3 ratio of donor to recipient bacterial cells. 10 µl of this mix was spotted on a dry LB agar plate without antibiotics nor DAP for overnight conjugation at 37 °C. The next day, the lawn of conjugation mixture was resuspended in 2 ml of PBS. 100 µl of this mixture and 100 µl of tenfold dilutions were plated onto selective agar plates containing 50 μg/ml kanamycin, the antibiotic marker tagging the *pgl* locus and incubated overnight at 37 °C.

**Table 2:** List of plasmids used in this study

Plasmid	Reference
pACYC*pgl*	[24]
pACYC*pglB*::km	[24]
pSEC*pgl*	[13]
pEXT21*pglB*	Timothy Scott, Wren Lab
pEXT20-SodB	[26]
pEXT20-*FlpA*	[26]
pEXT20-*G-NetB*	[13]
pMAD-*G-ExoA*	[37]

Single colonies were picked and grown in selective media (LB with 50 μg/ml Kan) overnight at 37 °C. Kanamycin resistant colonies were confirmed for presence of the plasmid by PCR amplification of the *pgl* insert. Colonies that were positive by PCR were picked and grown overnight at 37 °C, 180 rpm in 5 ml LB medium with 50 μg/ml Kan. Cultures that grew overnight were centrifuged for 2 min at 10,000×*g*. The pellets were washed 3 × in PBS and resuspended in 2 ml of PBS. 50 μl of the resuspension was spotted or streaked onto selective sucrose plates (15 g/l bacterial agar, 10 g/l tryptone, 5 g/l yeast, 15% v/v sucrose) with 50 µg/ml Kan, but in the absence of ampicillin to permit loss of the vector but retention of the kan^R^-marked *pgl* region. Kanamycin and sucrose resistant, ampicillin sensitive clones, were analysed for genomic insertion at the correct site by PCR. Double recombination events that resulted in kanamycin-resistant, sucrose-resistant, and ampicillin-sensitive colonies were verified by PCR with oligonucleotides 1&2, 3&4 and 5&6 described in Table 3 (Fig. 1C). Oligonucleotides 1 and 6 align to regions of the SDB1 genome upstream and downstream of the left and right homology arms used, respectively. Oligonucleotide 2 aligns to the beginning of the *pgl* locus, while oligonucleotide 5 aligns to the Kan cassette that tags the locus, thus amplicons obtained with primers 1&2 and 5&6 (∼1 kb) will only be present in the case of successful integration of the *pgl* locus at the chosen site. Oligonucleotides 3&4, instead, amplify the middle of the *pgl* locus and were used as a proxy to confirm integration of an intact *pgl* locus.

**Table 3:** List of primers used in this study

Primer name		Sequence (5′–3′)
1	fw	TGAGATCGAAAAGCAGCTGC
2	rev	TCCAAAGTGCCGTGGTTTTG
3	fw	GCCGCAAGATGAATACACGC
4	rev	CAAGCCCATGACCACTAGCA
5	fw	TGCCTGCTTGCCGAATATCA
6	rev	CCCAGTACATGTTCAGCAATG
pglBFw	fw	AAGAATTCATGTTGAAAAAAGAGTATTTAAAAAACCC
pglBRev	rev	AAGGATCCTTAAATTTTAAGTTTAAAAACCTTAGC
Fw*Pgl*B	fw	ACCACTCCGTTGCTAAGATAAA
Rev*PglB*	rev	GCCCGCTAGAATGTCTTTGA
gapAFw	fw	CTCCGCTGGCTAAAGTTATCA
gapARev	rev	CAGTCAGTTTGCCATTCAGTTC
rpoSFw	fw	GATGTGAATCGGCAAACGAATAG
rpoSRev	rev	CCGGATGATCGAGAGTAACTTG

Restriction sites are underlined

### Modification of carriers as acceptors for glycosylation

The proteins, superoxide dismutase (SodB) and fibronectin-binding protein (FlpA) from *C. jejuni*, detoxified necrotic enteritis toxin β (NetB) from *Clostridium perfringens*, and detoxified *Pseudomonas aeruginosa* exotoxin A (ExoA) were chosen as glycan acceptors. SodB and FlpA have been reported to reduce *C. jejuni* colonisation in poultry in previous studies [35, 36], NetB was shown to reduce necrotic enteritis in chickens [28], and ExoA, has been used previously in a successful *Francisella tularensis* vaccine study [29, 37]. Proteins G-SodB and G-FlpA were engineered to be PglB substrates as described in Vohra et al. [26]. All proteins were modified to ensure transport to the periplasmic compartment of the cell by addition of an *N*-terminal PelB signal peptide, apart from G-ExoA which contains a DsbA signal. All proteins contain a polyhistidine tag for affinity purification. G-NetB was modified to contain 5 DQNAT glycotags at both the *C*-and *N*-terminus [13] and detoxified by introducing the mutation W262A [38]. Finally, G-ExoA, a detoxified protein (L552V, ΔE553) contains two internal modifications that allow glycosylation of the protein by PglB, as well as containing four glycotags at the *N*-terminus and an additional 4 glycotags at the *C*-terminus [37].

### Transformation of SDB1 with carriers pEXT20-SodB, pEXT20-FlpA, pEXT20-G-NetB*, *pMAD-G-ExoA and with pACYC*pgl* containing the *pgl* locus

*Escherichia coli* strain SDB1, which lacks the function of wecA (ensuring the polysaccharide is built on undecaprenol with a bacillosamine instead of the preferential *N*-acetylglucosamine) and *waaL* (ensuring that no polysaccharide is transferred to lipid A) was used as the host strain for protein expression and glycoconjugate production. SDB1 was made electrocompetent and transformed with plasmids pEXT20-SodB, pEXT20-FlpA, pEXT20-G-NetB *and* pMAD-G-ExoA (all ampicillin resistant) encoding for the glycosylatable proteins, as well as pACYC*pgl* encoding for the *pgl* locus and pACYC*pglB*::km, encoding the *pgl* locus where PglB is non-functional owing to insertion of a kanamycin resistance cassette (chloramphenicol resistant and chloramphenicol and kanamycin respectively) [39]. These constructs enabled the expression of the glycoconjugates and their unglycosylated versions. The transformants were selected on LB agar supplemented with 100 µg/ml ampicillin and 30 μg/ml chloramphenicol for the glycosylatable proteins, and for the unglycosylated proteins at 100 µg/ml ampicillin, 30 μg/ml chloramphenicol and 50 μg/ml kanamycin and incubated at 37 °C overnight [29].

### Transformation of SDB1*pgl* with carriers pEXT20-SodB, pEXT20-FlpA, pEXT20-G-NetB and pMAD-G-ExoA

Strain SDB1*pgl*, was transformed with carriers pEXT20-SodB, pEXT20-FlpA, pEXT20-G-NetB *and* pMAD-G-ExoA to assess and compare the glycosylation potential of the *pgl* locus on the chromosome versus the *pgl* locus on a plasmid. SDB1*pgl* strain was made electrocompetent and transformed with 200 ng of pEXT20-SodB, pEXT20-FlpA, pEXT20-G-NetB or pMAD-G-ExoA. The transformants were selected on LB agar supplemented with 100 µg/ml ampicillin and incubated at 37 °C overnight.

### Generation of glycoconjugates using SDB1*pgl, *SDB1pACYC*pgl *and SDB1pACYC*pglB*::km

For all experiments, *E. coli* SDB1*pgl* was cultured in Luria–Bertani (LB) broth (Fisher Scientific, UK) supplemented with ampicillin (100 μg/ml) and kanamycin (50 μg/ml). Briefly, SDB1*pgl* pEXT20-SodB, pEXT20-FlpA, pEXT20-G-NetB or pMAD-G-ExoA strains were grown overnight at 30 °C, 37 °C and 42 °C. The next day, the cultures were diluted 1/100 and allowed to grow at 30 °C, 37 °C and 42 °C until an OD_600nm_ of 0.5 was reached. At that point 0.2% l-arabinose was added to SDB1*pgl* pMAD-G-ExoA, with a 0.2% l-arabinose supplementing addition 4 h later. 1 mM IPTG was added to SDB1*pgl* pEXT20-SodB, pEXT20-FlpA, pEXT20-G-NetB. All cultures were grown for a further 16 h.

For all experiments, *E. coli* SDB1 pACYC*pgl* and SDB1 SDB1pACYC*pglB*::*km* [39] were cultured in LB broth supplemented with ampicillin and chloramphenicol at 100 μg/ml and 30 μg/ml, respectively. Briefly, transformants of *E. coli* SDB1 pACYC*pgl* or SDB1 pACYC*pglB*::*km* carrying *pEXT*20-SodB, pEXT20-FlpA, pEXT20-G-NetB or pMAD-G-ExoA were grown overnight at 30 and 37 °C. The next day, the cultures were diluted 1/100 and allowed to grow at 30 and 37 °C until an OD_600nm_ of 0.5 was reached. At that point 1 mM IPTG was added to SDB1 pACYC*pgl* pEXT20-SodB, SDB1 pACYC*pgl* pEXT20-FlpA, SDB1 pACYC*pgl* pEXT20-G-NetB for induction of glycosylatable carriers. To SDB1 pACYC*pgl* pMAD-G-ExoA 0.2% L-arabinose was added for induction of the acceptor protein. All cultures were grown for a further 16 h at either 30 or 37 °C.

### Generation of glycoconjugates using SDB1*pgl* with additional inducible PglB

The strains SDB1*pgl* containing pEXT20-SodB, pEXT20-FlpA, pEXT20-G-NetB or pMAD-G-ExoA were transformed with pEXT21*pglB* to increase the copy number of the OST-encoding gene and discern if PglB was the limiting factor in the reaction. pEXT21*pglB* was generated by amplifying *pglB* from pACYC*pgl* using primers PglBFW and PglBrev (Table 3) and cloned into pEXT21 at EcoRI and BamHI restriction enzyme sites (Timothy Scott, personal communication).

### Glycoconjugate purification for comparison and quantification

Bacterial cells from cultures induced for 24 h were harvested via centrifugation at 5300×*g*, 4 °C for 30 min and were resuspended in ice-cold lysis buffer (50 mM NaH_2_PO_4_, 300 mM NaCl, and 10 mM imidazole). Lysis, wash, and elution buffer pH’s were adjusted to 8 by addition of NaOH. Resuspended bacterial cells were subjected to five rounds of mechanical lysis using a prechilled Stansted High Pressure Cell Disruptor (Stansted Fluid Power Ltd., UK) under 60,000 psi (410 MPa) in continuous mode. The lysate was then centrifuged at 10,000×*g* at 4 °C for 60 min and the supernatant combined with Ni–NTA (Qiagen, Japan) resin for 1 h at 4 °C. This was followed by washing the column with 200 ml of wash buffer (50 mM NaH_2_PO_4_, 300 mM NaCl, 20 mM imidazole pH 8.0), subsequently, 2 ml of elution buffer (50 mM NaH_2_PO_4_, 300 mM NaCl, 250 mM imidazole pH 8.0) was used to elute the proteins of interest. The collected fractions were visualised by western blot, and the glycosylated protein fractions were pooled and concentrated using buffer exchange columns Vivaspin 2 (Vivaproducts, UK) into PBS, prior to quantification using a NanoDrop (ThermoFisher, UK). Quantification of both glycosylated and unglycosylated proteins was determined using extinction coefficient and the molecular weight calculated by ProtParam (Expasy); G-FlpA extinction coefficient 54,780 M^−1^ cm^−1^, Molecular weight (MW) = 49,042.09 Da, and G-SodB extinction coefficient 47,120 M^−1^ cm^−1^, Molecular weight (MW) = 28,878.43 Da, G-ExoA extinction coefficient 98,320 M^−1^ cm^−1^, Molecular weight (MW) = 76,042.02 Da and G-NetB extinction coefficient 52,830 M^−1^ cm^−1^, Molecular weight (MW) = 42,409.22 Da.

### Assessment of glycoconjugate production by western blotting

To assess protein expression and glycosylation levels, a simultaneous two-channel fluorescence-based western blot (Odyssey LI-COR, LI-COR Biosciences, Hamburg Germany) was used to analyse the purified elution fractions. Freshly eluted samples were resuspended in 2 × Laemmli buffer and heated at 95 °C for 6 min. Heated samples, and a PageRuler Plus Prestained Protein Ladder (Bioline, UK) were separated on a NuPAGE 10% Bis–Tris Gel Novex®, then transferred using the Invitrogen TM iBlot TM2 gel transfer device to a Hybond™-C Extra nitrocellulose membrane (Amersham Biosciences, UK).

The membrane was then incubated with PBS 0.1% tween for 1 h at room temperature. This was followed by 3 × 10 min PBS washes. The following step was the incubation of the membrane with the primary antibody (mouse anti-His antibody (1:10,000)) and biotinylated soybean agglutinin (SBA) lectin (1:4000) for 1 h, a lectin specific for the GalNAc motif of the *C. jejuni* heptasaccharide. Following incubation, the membrane was washed for 30 min with PBS. The membrane was then incubated with the secondary antibodies used at a concentration of 1:10,000. IRDye® 680RD Goat anti-mouse for detection of His-tagged recombinant proteins and IRDye® 800CW Strepavidin to detect glycosylation (Odyssey® LI-COR Biosciences, UK). Detection of fluorescent signal was carried out using a LI-COR imaging system.

### qRT-PCR *of pglB* at 30 °C, 37 °C and 42 °C in SDB1*pgl*

Overnight cultures of SDB1*pgl* were grown in LB medium supplemented with 50 μg/ml of kanamycin, at 30 °C, 37 °C and 42 °C. RNA was extracted as previously described [40], with the small modification that 2 µg of total RNA from each sample was treated with TURBO DNase (Invitrogen) according to the manufacturer’s instructions.

cDNA was generated from DNase treated RNA using superscript III kit (Invitrogen) using random hexamers and following the manufacturer’s instructions. 2 µl of each sample were used as template in a RT-PCR using SYBRGreen dye-based PCR amplification and detection system using ABI7500 Fast instrument (Applied Biosystems). Comparative expression of *pglB* in SDB1*pgl* was analysed against the expression of housekeeping genes *gapA* and *rpoS* and using the ΔΔCt method [22]. Amplification was carried out using the following primers at a final concentration of 500 nM Fw*PglB* and RvPglB for *pglB*, gapAFw and gapARev for *gapA*, and rpoSFw and rpoSRev for *rpoS* (Table 3). Triplicate samples from three separate biological replicates were analysed (*n* = 9), statistical analysis was performed using a t-test.

### In vitro glycosylation reaction

An in vitro glycosylation reaction was undertaken to assess the effect of temperature on PglB function. The assay was based on Jaroentomeechai et al. [21], modified in house (Elizabeth Atkins personal communication, manuscript in preparation). Briefly, cultures of SDB1*pgl*, SDB1 pACYC*pgl*, SDB1 pEXT20-SodB, SDB1 pEXT20-FlpA, SDB1 pEXT20-G-NetB or SDB1 pMAD-G-ExoA were grown in LB at 30 °C, supplemented with 50 µg/ml kanamycin, 50 µg/ml chloramphenicol and 100 µg/ml ampicillin, respectively. These cultures were then lysed using a high-pressure cell disruptor as above and chilled to be used as heptasaccharide donor, as well as acceptor proteins. To all reactions (described in Additional file 1: Table S1) extra PglB produced from SDB1 pEXT21 *pglB* was also added. The reactions were prepared and incubated at 30 °C, 37 °C and 42 °C overnight and the ability to transfer *C. jejuni* heptasaccharide to the acceptor proteins at different temperatures was assessed by western blot (as described above).

## Supplementary Information

**Additional file 1. **Additional Figures and Tables.

## Data Availability

All data generated or analysed during this study are included in this published article [and its additional files].

## Abbreviations

PGCT: Protein Glycan coupling technology
LAVs: Live attenuated vaccines
Bp: Base pairs
Kda: Kilodalton
GlcNAc: *N*-acetylglucosamine
GalNAc: *N*-acetylgalactosamine
Glc: Glucose
OD: Optical density
SBA: Soybean agglutinin
