# Supplementary material for: PglB function and glycosylation efficiency is temperature dependent when the pgl locus is integrated in the Escherichia coli chromosome
Source: Microb Cell Fact. 2022 Jan 5;21:6. doi: 10.1186/s12934-021-01728-7 (PMC8728485; doi:10.1186/s12934-021-01728-7)
Supplement: Supplementary file 1 — Additional file 1. Additional Figures and Tables. [file 12934_2021_1728_MOESM1_ESM.docx]

**Supplementary data**

**Table S1**- Table indicating the in-vitro glycosylation reaction components as well as the temperature at which each reaction was incubated. S30 lysate buffer buffer (10 mM Tris Acetate, 14mM magnesium acetate, 60 mM potassium acetate, pH 8.2), 10% DDM (w/v) (n-dodecyl-β-d-maltopyranoside).

|  | **Temperature of incubation** (°C) | **Acceptor**  **(μl )** | **Glycan donor (μl) from SDB1pACYC*pgl*** | **Glycan donor (μl) from SDB1*pgl*** | **PglB donor**  **(μl)** | **S30**  **(μl)** | **1M MnCl_2_ (μl)** | **DDM 10% (μl)** | **Total**  **(μl)** |
| --- | --- | --- | --- | --- | --- | --- | --- | --- | --- |
| G-ExoA | 30 °C  37 °C  42 °C | 80  80  80 | 0  300  0 | 0  0  300 | 0  20  20 | 900  580  580 | 10  10  10 | 10  10  10 | 1000  1000  1000 |
| G-FlpA | 30 °C  37 °C  42 °C | 80  80  80 | 0  300  0 | 0  0  300 | 0  20  20 | 900  580  580 | 10  10  10 | 10  10  10 | 1000  1000  1000 |
| G-NetB | 30 °C  37 °C  42 °C | 80  80  80 | 0  300  0 | 0  0  300 | 0  20  20 | 900  580  580 | 10  10  10 | 10  10  10 | 1000  1000  1000 |
| G-SodB | 30 °C  37 °C  42 °C | 80  80  80 | 0  300  0 | 0  0  300 | 0  20  20 | 900  580  580 | 10  10  10 | 10  10  10 | 1000  1000  1000 |


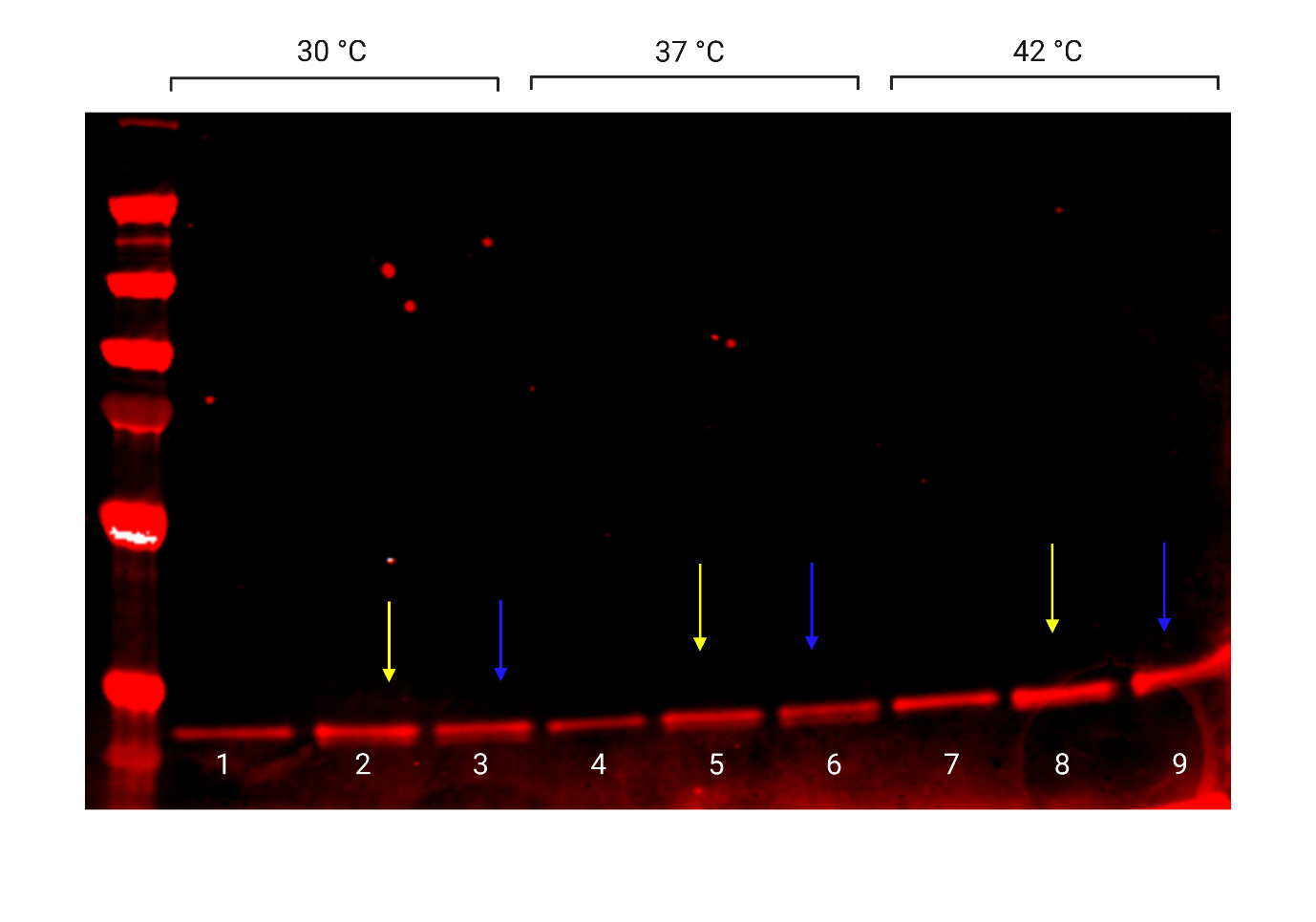


**Figure S1-** In vitro glycosylation reaction using carrier protein G-SodB. L-Protein ladder PageRuler plus (Bioline, UK) Lane 1, 4 and 7- negative control, G-SodB, no glycan donor incubated at 30°C, 37 °C and 42°C respectively; lane 2,5 and 8- G-SodB combined with glycan donor from SDB1pACYC*pgl* and SDB1pEXT21*pglB*, incubated at 30 °C, 37 °C and 42°C respectively; lane 3, 6 and 9- G-SodB combined with glycan donor from SDB1*pgl* and SDB1pEXT21*pglB* incubated at 30 °C , 37 °C and 42°C respectively. Yellow arrow indicates glycosylation when the donor glycan donor is from SDB1pACYC*pgl* and SDB1pEXT21*pglB* (lanes 2,5 and 8), blue arrow indicates glycosylation when donor glycan donor is from SDB1*pgl* and SDB1pEXT21*pglB* (lanes 3, 6 and 9).


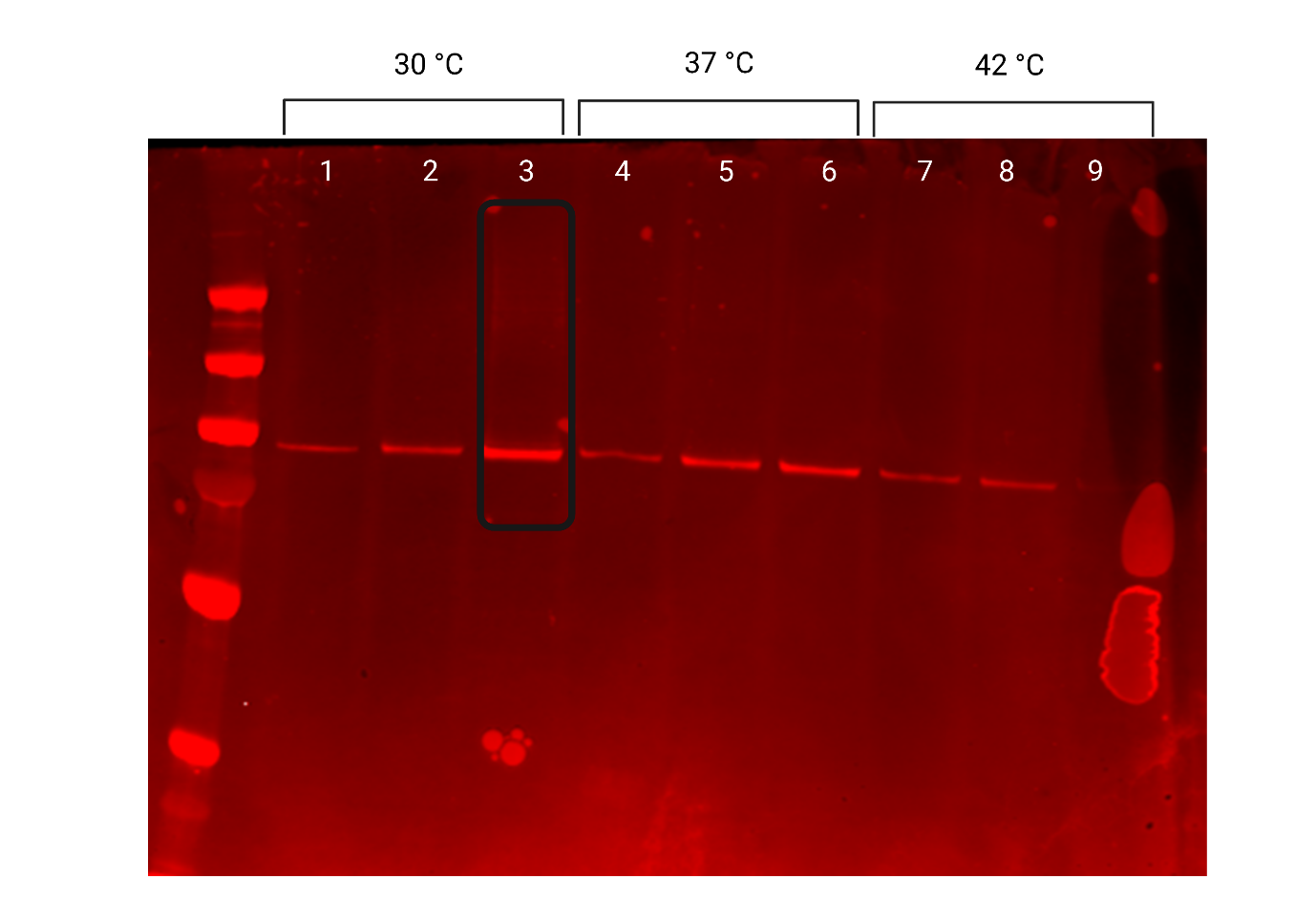


**Figure S2-** In vitro glycosylation reaction using carrier protein G-ExoA. L-Protein ladder PageRuler plus (Bioline, UK) Lane 1, 4 and 7- negative control, G-ExoA, no glycan donor incubated at 30°C, 37 °C and 42°C respectively; lane 2,5 and 8- G-ExoA combined with glycan donor from SDB1pACYCpgl and SDB1pEXT21pglB, incubated at 30 °C, 37 °C and 42°C respectively; lane 3, 6 and 9- G-ExoA combined with glycan donor from SDB1pgl and SDB1pEXT21pglB incubated at 30 °C , 37 °C and 42°C respectively. Black box indicates G-ExoA modified with glycan donor from SDB1pgl and SDB1pEXT21pglB (Lane 3).
